# A Classification Algorithm by Combination of Feature Decomposition and Kernel Discriminant Analysis (KDA) for Automatic MR Brain Image Classification and AD Diagnosis

**DOI:** 10.1155/2019/1437123

**Published:** 2019-12-30

**Authors:** Farzaneh Elahifasaee, Fan Li, Ming Yang

**Affiliations:** Department of Instrument Science and Engineering, School of SEIEE, Shanghai Jiao Tong University, Shanghai 200240, China

## Abstract

Magnetic resonance (MR) imaging is a widely used imaging modality for detection of brain anatomical variations caused by brain diseases such as Alzheimer's disease (AD) and mild cognitive impairment (MCI). AD considered as an irreversible neurodegenerative disorder with progressive memory impairment moreover cognitive functions, while MCI would be considered as a transitional phase amongst age-related cognitive weakening. Numerous machine learning approaches have been examined, aiming at AD computer-aided diagnosis through employing MR image analysis. Conversely, MR brain image changes could be caused by different effects such as aging and dementia. It is still a challenging difficulty to extract the relevant imaging features and classify the subjects of different groups. This paper would propose an automatic classification technique based on feature decomposition and kernel discriminant analysis (KDA) for classifications of progressive MCI (pMCI) vs. normal control (NC), AD vs. NC, and pMCI vs. stable MCI (sMCI). Feature decomposition would be based on dictionary learning, which is used for separation of class-specific components from the non-class-specific components in the features, while KDA would be applied for mapping original nonlinearly separable feature space to the separable features that are linear. The proposed technique would be evaluated by employing T1-weighted MR brain images from 830 subjects comprising 198 AD patients, 167 pMCI, 236 sMCI, and 229 NC from the Alzheimer's disease neuroimaging initiative (ADNI) dataset. Experimental results demonstrate that classification accuracy (ACC) of 90.41%, 84.29%, and 65.94% can be achieved for classification of AD vs. NC, pMCI vs. NC, and pMCI vs. sMCI, respectively, indicating the promising performance of the proposed method.

## 1. Introduction

AD, the world's supreme communal form of dementia, would be projected for flourishing in the coming years. Generally speaking, treatment for the disease would be too financially costly, with a very poorly understood cause; furthermore, there is no curative treatment up to now. However, MCI would be considered a transitional phase between age-related cognitive failing and AD. In particular, structural kind of MRI scans would be responsible for the information around interior anatomical structures and brain tissue morphologies similar to gray matter (GM), white matter (WM), and cerebrospinal fluid (CSF). Actually, early diagnosis of AD and its prodromal stage would be a significant reason for the possible delay of the disease; moreover, there is a great deal of interest in new methods developed for earlier detection of this dementia.

Generally, structural brain anomalies consider a sensitive disease feature that is observable on MR brain images, actually, one of several known biological markers of the disease. Machine learning techniques, in particular, based on the MR brain image are able to effectively handle the high-dimensional features, i.e., structural MR imaging, and thus permit the automatic AD classification [1]. However, this strategy is widely being studied in recent years [2–6]. In the previous studies, feature selection (FS) approaches for bioinformatics application have been moved from a descriptive instance to model building. Most papers explored are domains, which have up to tens of thousands of features that are not easy to work with.

In particular, due to the high dimensionality, the classification task of neuroimaging data would be a big challenge. Generally, the FS technique could be used for the supervised and unsupervised learning scenarios; however, in this paper, we are just attentive on learning classification of supervised problems. Classification difficulties would serve more often as a design than regression difficulties, and also only vectorial input data would be considered [6]. Recent research studies, which would be focused on characters with MCI, may have a conversion to the AD higher rates (amount 15% per year) than the individuals in NC [5]. Actually, there would be many investigator's study in which MCI would be very early to AD, as it has been demonstrated that numerous MCI beings have parallel patterns of the atrophy and b-amyloid deposition when suffering from AD [6–10]. Although some MCI people are clinically stable over time, some others may have temporary normal brain structures [7].

Nowadays, some researchers employed deep learning for classification of neuroimaging. However, through deep learning, it tries to fit the model by going deeper inside the training set, and overfitting happens [8]. Generally speaking, given conversion from MCI to AD high rates and also plentiful neuropathology previously evident in the postmortem of the MCI [9, 10], superior emphasis would be placed on recognizing those NC individuals who extant developing AD-like brain atrophy patterns, which could be comparatively more than the progress of MCI to AD [11]. As a matter of fact, documentation of such peoples at an initial phase beforehand symptoms of clinical beginnings might lead to being in more actual pharmacological treatment intervention for AD as those become accessible [12, 13]. Consequently, here, we proposed a classification algorithm of MR brain images by a combination of feature decomposition and KDA for MCI/AD classification.

## 2. Similar Studies

Generally speaking, some normal aging longitudinal research studies have measured the brain variations over the regions of interest (ROI) and also voxel-based analysis [7–20]; moreover, they have consideration greater than before about how dissimilar the regions of brain alteration in the normal aging populations are. We can say that although the entire brain or interest volumes regions would be concentrated with the aging and AD, their individual differences and the intersection through populations reduce accuracy of disease diagnosis through individuals, particularly at the initial stages of the disease.

There was a great-dimensional pattern classification approaches development in the past years [7, 21–23] so that suggestions are impending for achieving greatly sensitive and also exact biomarkers of neuroimaging from the individuals, instead of groups, that has huge significance for the premature diagnosis and also for the management of the individual patient. In general, these approaches use algorithms related to sophisticated pattern analysis which would be trained to recognize normal patterns or abnormal structures and also function [22], which are used to classify at the individual level. Actually, researchers have depicted previously which brain atrophy three-dimensional patterns discriminate NC and AD with high accuracy [7]. Actually, in most of the classification techniques, the two following steps were included: (1) selection and discriminative features extraction from the original neuroimaging data and (2) optimal separating of prime hyperplane learning in the great-dimensional feature space performing AD classification [5]. One technique that could be used vastly would be partitioning of the MR brain image into multiple anatomical regions, in other words, ROIs, from side-to-side labeled atlas warping [2–5].

Generally speaking, for selecting the most discriminative features, the selection of a discriminative multitask feature process would be crucial. Marginal Fisher analysis was combined with norm-based multikernel learning for obtaining the sparsity of ROIs, which guide to concurrently select the subset of the relevant brain regions and also learn dimensionality transformation [13] In addition, to the features of ROI, deep learning networks were employed for the hidden huge-level features extraction from the ROI measurements for the classification of AD [2, 3, 20]. In [3], the authors proposed using a stacked autoencoder for the hidden huge features from ROI learning for the improvement of classification performance. Even though promising outcomes have been reported for analysis of the brain image, there would be still some limits in ROI constructed approaches.

Firstly, ROI explanation demands the accumulation of works and the researcher's long-term experience. Secondly, abnormalities in morphology caused by AD do not every time happen in the predefined ROIs, and also they may include multiple ROIs or extracted ROI part; in this way, predictable and performance might not be very constant. Actually, in the previous years, on the one hand, numerous huge-dimensional classification approaches have been proposed for automatically discriminating patients with AD or MCI and also NC based on T1-weighted MRI brain images. On the other hand, these approaches were evaluated on the same features (for instance, all the features would be class-specific components or maybe they are a non-class-specific component). For example, Moradi et al. [24] used the semisupervised scheme for making use of data that are unlabeled for training characteristically not much of labeled data with so many unlabeled data and could classify MR brain images with the low-density separation (LDS) method on semisupervised data.

Moreover, another researcher [13] had used cortical thickness features and by applying principle component analysis-linear discriminant analysis (PCA-LDA) succeeded to obtain a sensitivity of 63% and a specificity of 76% on a group of 72 pMCI and 131 sMCI patients mentioning that using this approach requires lots of time; Ye et al. [25] engaged AD and also NC subjects as the labeled data and also subjects of MCI as the unlabeled data, a likewise predicted disease labels for the MCI cases; the authors also used semisupervised classification. Problems through ROI features would be potentially addressed through the voxel-wise technique [22], in other words, extracting voxel-wise features for classification of the image [25].

In addition, the analysis of the voxel-wise image would be very sensitive toward the registration error and also the variability of noise large intersubject. Meanwhile, these limits would be relieved through smoothing the imaging features through employing any Gaussian filter; smoothing would be considered usually complete on the brain entirely for all subjects uniformly and additionally, therefore, would be not adaptive for the anatomical structures, shapes, and abnormal regions.

More essentially, the number of voxel-wise imaging features from the whole brain would be often very enormous (in other words, the millions) even though the training sample numbers would be very small (in other words, in hundreds) in the neuroimaging field of research. This could create a classification technique easy to overfitting on the training set and moreover does not generalize fine to the test set. Therefore, how to extract the discriminative features and classify the imaging features of high dimension through an imperfect subject number would be still challenging for MR brain image analysis in AD diagnosis.

## 3. Methodology

**Figure 1:**
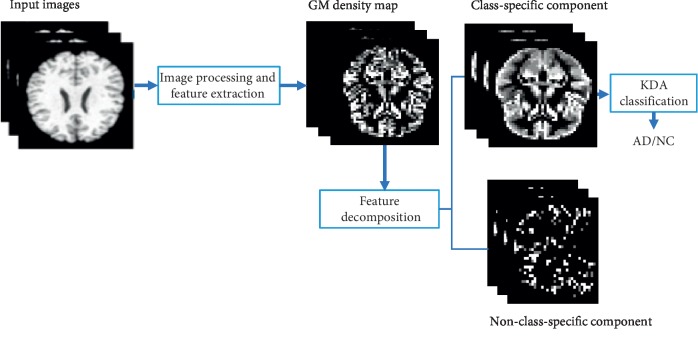
Classification of proposed method.

This section was divided into three parts, where the first section is about MR brain image preprocessing and feature extraction that should be done in all MR brain imaging. Subsequently, feature decomposition and how we divide entire brain images to class-specific features (features with label) and non-class-specific features (features without labels) are explained in detail, mentioning that these two groups of features are achieved by the dictionary learning algorithm; in the end, this part ends with an MR brain image that was applied with the feature decomposition algorithm. Finally, in Section 3.3, we explained the **c**lassification of AD through KDA and clarified KDA would be applied for mapping original nonlinearly separable feature space to linearly separable ones. Lastly, the aforementioned selected features are classified by the nearest neighbor (NN) technique.  Figure 1 shows the proposed algorithm.

### 3.1. Image Processing and Feature Extraction

In this work, MR brain images of T1-weighted would be used for testing our proposed method. In this paper, we used Matlab software, statistics, and machine learning toolbox for implementation. Though the proposed algorithm makes no statement on any class-specific neuroimaging modality, the MR imaging would be extensively obtainable, noninvasive, and moreover often be used as the first biomarker in brain diseases difference diagnostics caused by the memory problems. At this work, MR imaging data of T1-weighted would be tested, aiming at demonstrating our proposed performance technique. Generally, in Alzheimer's disease neuroimaging initiative (ADNI) [26], MR imaging datasets comprise standard MR images of T1-weighted attained sagittal using the volumetric 3D magnetization prepared rapid gradient echo (MPRAGE) through 1.25 × 1.25 mm in-plane three-dimensional resolution and 1.2 mm dense sagittal slices.

Note that most of these images would be achieved with the 1.5 T scanners, and a few were developed with the use of 3 T scanners. Comprehensive information about MR brain images gaining procedures would exist at the website of ADNI. The current paper comprises baseline MR brain images of 229 NC subjects, 198 AD patients, and 403 MCI cases (comprising 236 sMCI and also 167 pMCI subjects). The MR brain images are first preprocessed according to the previous validation [3]. Precisely, inhomogeneity of intensity on the T1-weighted MR brain images would be corrected through means of the nonparametric nonuniform intensity normalization (N3) algorithm [26]. At that moment, an automated and strong skull stripping technique would be applied for brain extraction and cerebellum removal [27]. Generally, each brain image would be additionally segmented obsessed with three tissue volumes types, for instance, WM, GM, and also CSF volumes. Entire tissue volumes would be normalized spatially together on a standard space through an algorithm of mass-preserving deformable warping [28].

In brief, warped mass-preserving tissue volumes reproduce tissues spatial distribution in the innovative brain. Therefore, we call these tissues of warped mass-preserving volumes as density maps of tissue in this paper. Meanwhile, GM would be more related to the AD and MCI cases than WM and CSF, voxel-wise GM densities would be used as imaging features to demonstrate the classification performance of our proposed method. Given *M* training images, with each represented by a feature vector and a respective class label, the brain classification involves the selection step in most relevant features furthermore by decoding step disease conditions like the class labels.

During warping of images, the volume of tissue in any region size would be preserved; in other words, it would be improved if the region is compressed, and vice versa. As a matter of fact, the tissue volume of the warped was mass-preserving that would replicate the distribution of the three-dimensional distribution in the imaginative brain. Actually, we would call the volume of the tissues as tissue density maps in current work. Meanwhile, GM would be more connected to the AD than CSF or WM, and densities of the voxel-wise GM are employed as features of imaging for classification of the testing proposed technique performance of ours. Note that only the training part of features is used for the decomposition and classification.  Table 1 demonstrates the demographic characteristics of the studied subjects from the ADNI dataset.

### 3.2. Feature Decomposition

As a matter of fact, whole brain GM densities and voxel-wise densities that are extracted in the above section would be of huge dimension, which includes both noninformative and informative features, and one way to make classification of disease easy would be by using some techniques such as the *t*-test to identify discriminative information from the whole brain moreover description of result.

However, the extracted features were despoiled by some common factors such as image capturing and processing. Besides, the variations of these features for different subjects may be larger than those caused by disease, which results in large intraclass variations. These problems will cause a reduction in discrimination for the features. In this work, we propose a decomposition of the GM density features into class-specific and non-class-specific components based on dictionary learning and using only class-specific components with discriminative information for further classification. GM densities of the whole brain are concatenated into a column vector *x*; suppose there would be *N* samples of training {*x*_1_,…, *x*_*n*_,…, *x*_*N*_} which fit to the *C* classes, represented by **X**=[*X*_1_,…, *X*_*l*_,…, *X*_*C*_] ∈ *ℜ*^*M*×*N*^, wherever *N*=*N*_1_+⋯+*N*_*l*_+⋯+*N*_*C*_ and also *X*_*l*_ ∈ *ℜ*^*M*×*N*_*l*_^ consists of *N*_*l*_ training samples belonging to the *l*-th class (mentioning that *C*=2 in this work). Except it is noted especially distinguished, and entire feature vectors in this work would be represented through column vectors; moreover, ‖·‖_2_ means standard Euclidean norm although ‖·‖_1_ signifies standard L1 norm. In general, test data *y* could be represented through any linear training sample combination from entire categories as follows:

(1) $$\begin{array}{c} y=X\alpha +\varepsilon , \end{array}$$

where *α* is the coefficient vector, which is associated with the training samples and *ε* is the approximation error. For a perfect representation, a sparsity constraint is often put on the coefficients *α*. Note that if rank (*y*) = *M* = *N*, we would have a distinctive perfect illustration *α* which leads to *e* = 0 on behalf of any query image *y*. Moreover, this could be realized that there exists a representation *α*_*i*_ leading to *e*_*i*_ = 0 for any class as long as its training samples are not linear dependent and *n*_*i*_ ≫ *m*.

If the dictionary *X* is overcomplete, i.e., *n* > *m,* the perfect representation *α* is not unique; i.e., there are an infinite number of solutions *α* that lead *e* = 0. Sparse coefficients optimization makes the samples of training compete against each other for achieving its test image representation. The sparse representation of discriminative nature can be used for classification, i.e., the sparse representation-based classification (SRC). However, representation features comprise both data of class-specific and also non-class-specific information. Thus, coefficients of nonzero that consider sparse may be related to the multiple classes. If representation features can be decomposed into class-specific, non-class-specific components and class-specific components of features will have a better discriminating ability for classification. The feature decomposition is as follows:

(2) $$\begin{array}{c} y=A\alpha +B\beta +\varepsilon , \end{array}$$

where *A* denotes representation of class-specific components and *B* denotes components of non-class-specific representation. This can be achieved by dictionary learning with training data. During the training process, the training samples *X* will be decomposed into the class-specific and non-class-specific dictionaries for better discriminative representation as follows:

(3) $$\begin{array}{c} x_{n}=A\alpha_{n}+B\beta_{n}+\varepsilon_{n}. \end{array}$$

To solve the above decomposition problem, we assume that *α* is an identity matrix, and the regularization of the dictionary decomposition would be modeled as follows [29]:

(4) $$\begin{array}{c} \begin{array}{cc} \underset{A,B,\beta ,E}{\min} & {\left\|A\right\|}_{*}+\mu {\left\|B\right\|}_{*}+\gamma {\left\|\beta \right\|}_{F}^{2}+\tau {\left\|E\right\|}_{1},\\ \text{s}.\text{t}.\; & X=A+B\beta +E, \end{array} \end{array}$$

where *μ*, *γ*, and *τ* would be parameters for balancing four terms minimization, ‖·‖_*∗*_ denotes the nuclear norm, and the squared Frobenius norm ‖*β*‖_*F*_^2^ would be a summation of squared *L*_2_-norms, in which *β* would be a constant aimed at a negotiation amongst *α* sparsity and representation error ‖*e*‖_2_^2^. Moreover, the squared Frobenius norm ‖*β*‖_2_^2^ would be squared *l*_2_ norms sum and *τ* and *λ* would be parameters for balancing the minimization of four terms. Regularization of decomposition could be solved through minimizing two of the four unknowns through fixed others iteratively as in [30]. Through the above decomposition of the dictionary, we not only learned a class-specific dictionary *A* moreover any non-class-specific dictionary *B* with training data but also alleviated the other corrupted training data errors since the training data *E* random sparse noise would be removed from two learned dictionaries. For a test data *y*, *α*, *β*, and *ε* could be achieved by solving below optimization obstacle:

(5) $$\begin{array}{c} \begin{array}{cc} \underset{\alpha ,\beta ,\varepsilon }{\min} & {\left\|\alpha \right\|}_{1}+\gamma {\left\|\beta \right\|}_{2}^{2}+\tau {\left\|\varepsilon \right\|}_{1},\\ \text{s}.\text{t}. & X=A\alpha +B\beta +\varepsilon . \end{array} \end{array}$$

With the help of using augmented Lagrange multiplier (ALM), the above optimization difficulty could be solved [29, 30]. After decomposition, the class-specific component consisting of discriminative information would be used for classification, while the non-class-specific component will not be considered. As mentioned above, optimization difficulty could be solved through the ALM technique as in [31]. After decomposition, the class-specific component consisting of discriminative information is used for classification, while the non-class-specific component will not be considered.

**Figure 2:**
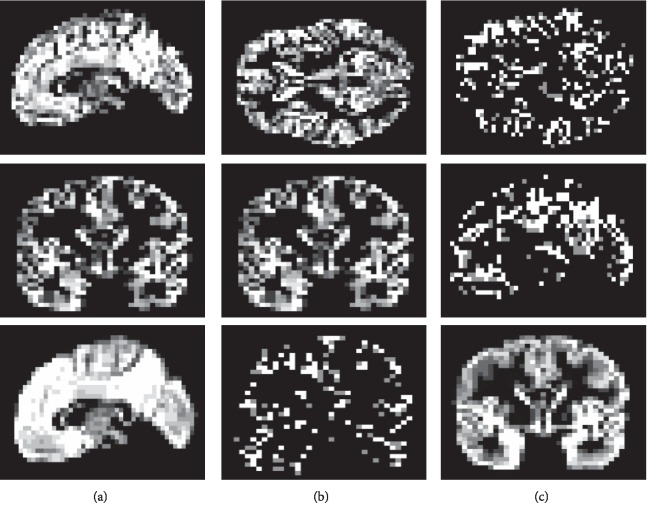
Decomposition of (a) a sample input GM density map into (b) the component of non-class-specific (without labeled features) and (c) component of class-specific (labeled features).

For illustration, Figures 2(a)–2(c) show feature decomposition results of a sample GM density map, the non-class-specific component, and also the class-specific component, respectively. It should be mentioned that the training data *X* will be decomposed into the class-specific and non-class-specific dictionaries *A* and *B* by dictionary learning with training data during the training process (actually, *A* and *B* are considered as two dictionaries with labeled features and without labeled features, respectively). After training, the test set is also decomposed by using *A* and *B*.

### 3.3. AD Classification by Kernel Discriminant Analysis

The decomposed class-specific features (labeled features) from entire brain densities of GM would be still of enormous dimensionality, compared to minor subject numbers, that could create a classification of the disease problematic. KDA applies the nonlinear function of the kernel to map the high-dimensional data against the space of nonlinear discriminant which can overwhelm the variation of interclass and also would maximize gap that exists amongst images from dissimilar subjects. As a matter of fact, the discriminative analysis would be assumed as the supervised arrangements for classification and also feature extraction; moreover, technique would be mainly used for unsupervised scenario wherever distributions of the underlying class are situated separated in the best criticism. On the contrary, in the real case due to a solution number, this goal is not fulfilled.

It would be probable realizing any optimal representation through spending computational power or a certain time. Nevertheless, with the limited considering time and also resources, it would be normally incredible to account for the entire possible feature linear combinations. Other researchers [31, 32] recommended using a sparse principal component algorithm (SPCA) through the use of the “elastic net” framework for regression of *L*_1_ penalized on ordinary PCA which was solved so professionally by employing least-angle regression (LARS) [33, 34]. Other researchers in [14, 33–36] proposed spectral limits outline for sparse to the subspace learning. Predominantly, it can be proposed for both greedy and exact algorithms for sparse LDA and PCA. Actually, the kernel projective function subspace learning would be written as below:

(6) $$\begin{array}{c} f\left(x\right)=\alpha^{T}K\left(:,x\right)=\overset{m}{\underset{i=1}{\sum }}\left[\alpha_{i}K\left(x_{i},x\right)\right]. \end{array}$$

Commonly speaking, at normal subspace learning of the kernel algorithm, *α*_*i*_ mostly would be considered nonzero. Moreover, the projective function would be reliant on entire samples which belong to the training set. From this aspect, the algorithm of sparse kernel subspace learning would share the same support vector machine (SVM) knowledge [33, 34]. Moreover, these with nonzero *α*_*i*_ samples could also be called support vectors. Generally speaking, one of the benefits would be that it requires less loading time, and obviously, fewer amount of time is the required amount of time which considered computational demanded for the testing period. Following [34–36], sparse regression KDA (SRKDA) could be shown as follows:

(7) $$\begin{array}{c} \text{Max}\frac{\alpha^{t}KWK\alpha }{\alpha^{t}KK\alpha }\text{ subjecting to the card}\left(\alpha \right)=K. \end{array}$$

The possible set would be all sparse *α* ∈ *R*^*m*^, that is, with the *k* basics of nonzero also cad (*α*) as their *L*_0_-norm. This optimization obstacle would not be so easy for solving as it would be nondeterministic polynomial (NP) hard. Previous researchers in [14, 33, 34] recommended any spectral bound framework for the learning of sparse subspace. Mostly, they advocated both exact algorithms and also greedy algorithms for sparse PCA moreover sparse LDA. Actually, their bounds of the spectral framework would be grounded on following perfect sparse solution condition. It is defined as *A* = *KWK* and also *B* = *KK* for the sake of ease. Any vector of the sparse *α* ∈ *R*^*m*^ with cardinality *k* yielding objective value of that would be maximum in equation (7) that leads to that

(8) $$\begin{array}{c} \lambda_{\max}=\frac{\alpha^{T}A\alpha }{\alpha^{T}B\alpha }=\frac{\beta^{T}A_{k}\beta }{\beta^{T}\beta_{k}\beta }. \end{array}$$

Generally speaking, *β* ∈ *R*^*k*^, where *k* is the components of nonzero in the *α* and also *k* × *k* submatrices of principle which are related to *A* and also *B* achieved through removing rows and columns approaching toward zero *α* indices.

It is worth mentioning that the *β* quadratic form that is assumed to be *k* dimensional would be equal toward a standard unconfined generalized Rayleigh proportion, which could be answered through eigenproblem generally. The precise algorithm for subspace learning could be attained by the overhead observation: discrete research for *k* indices creates subproblem (*A*_*k*_, *B*_*k*_)*λ*_max_ maximum though this observation owns no well-organized algorithm. The reason for that would be because a comprehensive search would be still NP hard. Some previous researches [14, 33, 34] proposed an effective greedy algorithm for solving this obstacle which associates background removal and also forward selection [14, 33–35]. Moreover, as KDA uses the Lasso regularization technique because of *L*_1_ penalty natural surroundings, some of the coefficients *α*_*i*_ would be shrunk for exaction zero if *δ* would be considered large enough. Therefore, we could conclude that could be what we require exactly.

Dissimilar from SRC or LDA which solitarily uses the distance of *L*_2_-norm, KDA could exploit dissimilar distance measures with the kernel functions for also dissimilar features. Through selecting the suitable kernel, KDA performance would be enhanced than LDA and SRC. In this work, we employ a discriminative spectral regression kernel analysis (SRKDA) that anticipated in for the final classification due to its computational efficiency. The Gaussian kernel would be selected in our experiments. SRKDA performs the discriminant analysis by use of spectral graph analysis and regularization [37–39].

## 4. Results

In brief, here, we would first present ADNI datasets of images moreover the implementation of our proposed method; afterward, we would have a discussion of our result, and finally, we compare our method with the earlier techniques.

### 4.1. Experimental Results

Firstly, it is a brief information about participants in Table 1 based on their number, age, gender, and MMSE (mini-mental state examination). At that point, we would extant the extensive experiments for testing the proposed technique on the pMCI vs. NC and AD vs. NC and also classification of pMCI vs. sMCI; moreover, we would in addition do comparison of our proposed scheme with the other procedures that can be found in the various available literatures and also make some discussion. Actually, in this section, we would indicate the extensive experiments performed for testing the proposed classification process on pMCI vs. NC and AD vs. NC and also pMCI vs. sMCI classification. As a matter of fact, this technique on T1-weighted MR brain images of the ADNI database would be from 262144 features which include 830 subjects counting 198 patients of AD and also 167 pMCI, 236 sMCI, and 229 NC for evaluation.

In fact, the image processing would be conducted for the feature extraction as illustrated on image processing and feature extraction section. The MR brain images are automatically cerebellum-removed and skull-stripped [34]. As declared before, all brain images would be further segmented to the 3 kinds of tissue, for instance, GM, WM, and also CSF volumes. Subsequently, an intensity inhomogeneity correction on the entire specific volume of tissues could be normalized spatially unmoved against normal space (known as stereotaxic space) through the algorithm of a mass-preserving deformable warping which was proposed in [39].

Volumes of warped mass-preserving tissue replicate three-dimensional tissue distribution in the innovative and basic brain. As a matter of fact, we can know these tissue density maps of warped mass-preserving tissue volumes here. It could be said that the volumes of the tissue that were three-dimensionally normalized would be known as tissue densities in the current paper; also here, we employ only the GM density map by imaging features because of its dependency more to the AD. To decrease the effects of noise, inaccuracy of registration, and also anatomical differences of interindividual, the GM density map would be further smoothed with the help of the Gaussian filter and downsampled from 256 × 256 × 256 to 64 × 64 × 64 voxels through a factor of 4. The voxel size is 4 × 4 × 4 mm. The downsampled GM density map would be employed for the feature decomposition and classification. This could decrease the computational and memory costs deprived of sacrificing accuracy.

In brief, for evaluating the performance of classification, cross-validation of 10-fold approach was employed for training and testing our proposed scheme and to decrease influences of random factors. Every time, only one dataset fold would be employed for the testing; meanwhile, the other folds would be employed for training. In our experimental outcomes, some performance measures of classification would be employed for the ACC for evaluation comprising sensitivity (SEN), specificity (SPE), and also the area under the curves (AUCs). Actually, ACC would be calculated by classifying correct subject amongst all populations. SEN would be calculated as the amount of correctly classified positive samples (AD subjects) amongst the samples of the positive total number. SPE would be calculated as the appropriately classified samples of negative (NC subjects) proportion amongst whole negative sample numbers.

The first experiment (Table 2) would be the result of comparison of the feature decomposition, KDA, and also the proposed technique for classification of pMCI vs. NC. For testing the feature decomposition performance, we employed the *L*_1_-regularized SRC on decomposed features for making the classification. For testing KDA performance, we directly apply the KDA on the GM densities deprived of feature decomposition for making the classification.  Table 3 demonstrates performance comparison through aforementioned schemes aimed at AD vs. NC classification.  Table 4 indicates classification performances comparison through these different methods for classification of pMCI vs. sMCI. Actually, Table 5 compare classification performances of three methods (LDA, SRC, and SVM) with the proposed method ACC for AD vs. NC case.

**Figure 3:**
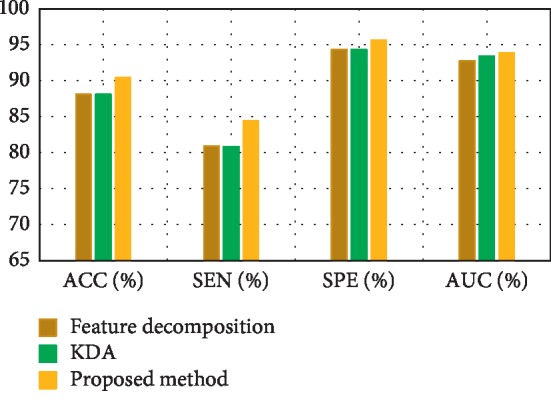
Comparison result of the feature decomposition, KDA, and the proposed method for classification accuracy of AD vs. NC.

**Figure 4:**
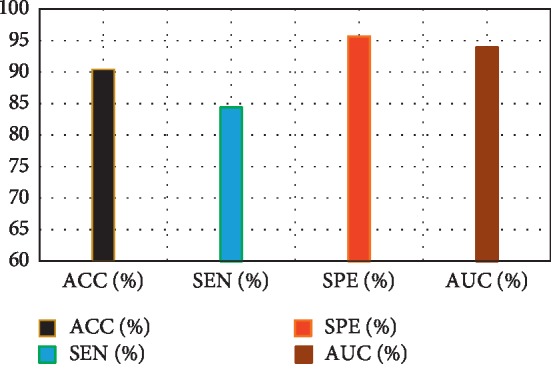
Proposed method results.

**Figure 5:**
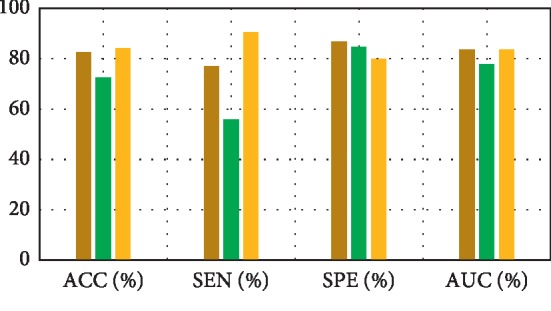
Comparison results of the feature decomposition of pMCI vs. NC.

Figure 3 depicts the comparison result of feature decomposition, KDA, and proposed a method for AD vs. NC classification. Note that all results in our paper have been tested on publicly available datasets based on ACC, SEN, SPE, and AUC.  Figure 4 shows the proposed method result based on ACC, SEN, SPE, and AUC parameters. Furthermore, Figure 5 shows the comparison results of the feature decomposition, KDA, and proposed method for classification of pMCI vs. NC cases. Since our MR image data are same as those in [5], the results of SVM and SRC in [5] are presented for comparison in Table 5. For LDA, we implement it through Matlab, statistics, and machine learning toolbox.

Figure 3 compares the classification accuracy for AD vs. NC with feature decomposition, KDA, and proposed technique. Moreover, Figure 4 shows classification accuracy by a proposed method based on ACC, SEN, SPE, and AUC parameters.

**Figure 6:**
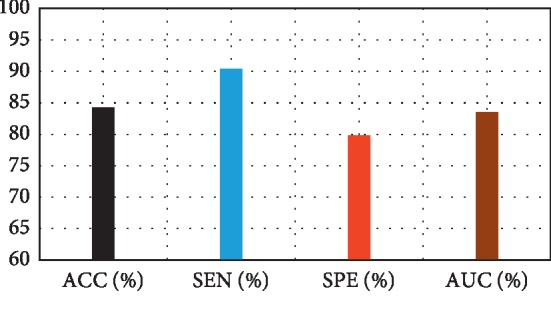
Proposed method results.

Figure 5 compares pMCI vs. NC classification based on ACC, SEN, SPE, and AUC parameters by feature decomposition, KDA, and proposed a method, while Figure 6 shows the proposed technique classification based on ACC, SEN, SPE, and AUC parameters.

**Figure 7:**
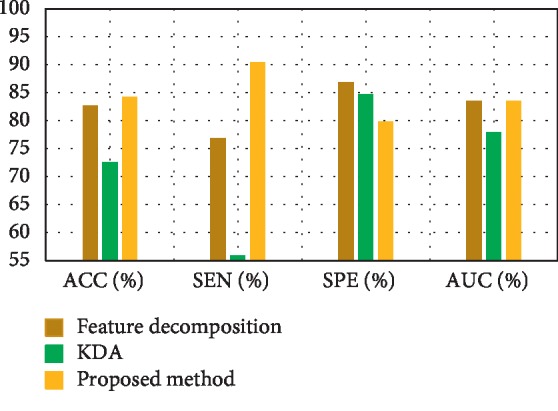
Comparison results of the feature decomposition, KDA, and also the proposed technique for classification of pMCI vs. NC.

**Figure 8:**
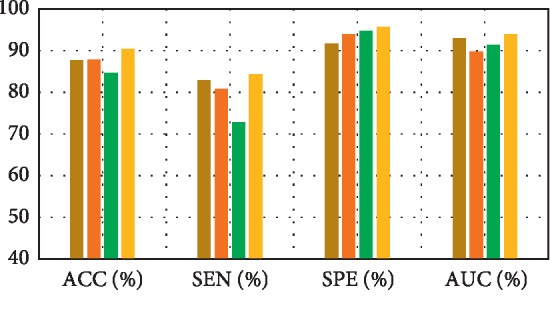
Results of proposed method comparison and other methods for AD vs. NC.

Figure 6 shows the pMCI vs. NC classification based on ACC, SEN, SPE, and AUC parameters. Furthermore, Figure 7 depicts the comparison results of the feature decomposition, KDA, and proposed technique for classification of pMCI vs. NC.  Figure 8 shows the results of proposed method in comparison with the other methods (LDA, SRC, and SVM) and with the proposed method for AD vs. NC.

**Figure 9:**
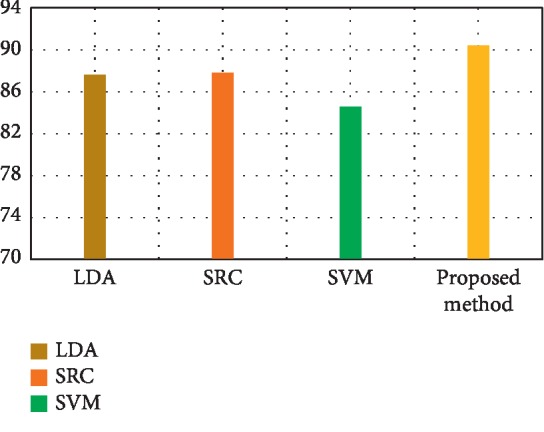
Diverse results of comparison.

**Figure 10:**
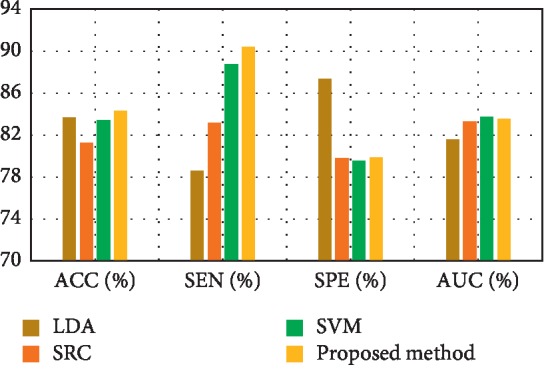
Results of proposed method comparison and other methods for pMCI vs. NC.

**Figure 11:**
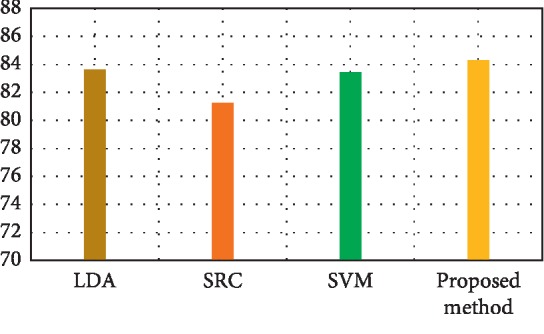
Results of proposed method comparison and other methods for pMCI vs. NC.

**Figure 12:**
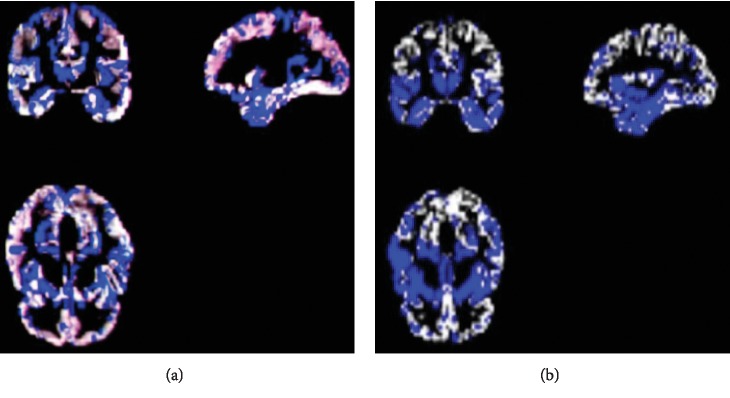
Identified biomarkers of GM density map by using the *t*-test (a) before and (b) after feature decomposition for AD vs. NC classification.

**Figure 13:**
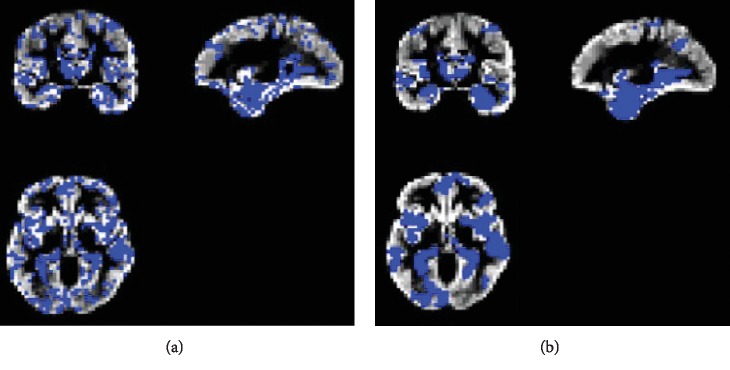
Identified biomarkers of GM density map by using the *t*-test (a) before and (b) after feature decomposition for pMCI vs. NC classification.

**Figure 14:**
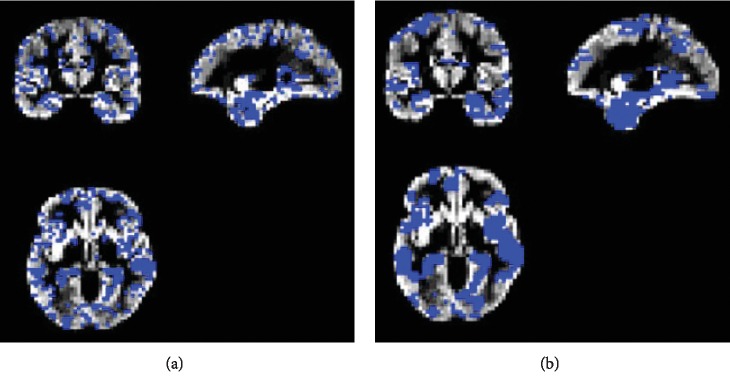
Identified biomarkers of GM density map by using the *t*-test (a) before and (b) after feature decomposition for pMCI vs. sMCI classification.

In addition, Figure 9 compares the results of the proposed method with the aforementioned methods for AD vs. NC classification results, while Figure 10 depicts the classification of pMCI vs. NC based on ACC, SEN, SPE, and AUC parameters and Figure 11 the results of the proposed method in comparison with the other methods (LDA, SRC, and SVM) for pMCI vs. NC. Moreover, Figure 12 shows identified biomarkers of GM density map by using the *t*-test (a) before and (b) after feature decomposition for AD vs. NC classification.  Figure 13 demonstrated identified biomarkers of the GM density map by using the *t*-test (a) before and (b) after feature decomposition for pMCI vs. NC classification, and Figure 14 illustrates identified biomarkers of the GM density map by using the *t*-test (a) before and (b) after feature decomposition for pMCI vs. sMCI classification.

Tables 6 and 7 compare the classification results to those of classification of existing approaches for pMVI vs. NC and also pMCI vs. sMCI. SRC was applied for AD classification in [5]. Since our MR image data are the same as those in [5], the results of LDA, SRC, and SVM_1_ reported in this paper are presented for comparison in Table 5. We applied all of implementation using the Matlab functions, statistics, and machine learning toolbox.

### 4.2. Discussion

Here, we would have evaluated the proposed method performance of classification with the 830 observations of MR images, developed from the ADNI study. This work proposes a new algorithm for the classification technique created from a combination of feature decomposition and KDA for automated classification of MR images for pMCI vs. NC, AD vs. NC, and pMCI vs. sMCI classification. SEN, SPE, and AUC parameters mention that all SVM that were used in this paper is radial basis (RBF) kernel SVM, while Figure 11 shows the proposed method classification accuracy for pMCI vs. NC.

Feature decomposition based on dictionary learning would be used to separate the class-specific components and non-class-specific components in the features, while the KDA would be applied for mapping imaginative nonlinearly separable features space to linearly separable one. Note that we have used brain MR images of the whole brain meanwhile by applying the *t*-test on features for selecting some features that were related to AD.

### 4.3. Results Comparison

Table 8 indicates a comparison between our proposed classification method results and the previous approaches. In general, Mistra et al. [40] attained high extrapolative enactment for the sMCI vs. pMCI classification. On the contrary, in the research study by Misra et al., datasets (27 pMCI and 76 sMCI) were so small compared to the existing researches which make the comparison not easy with the other researches. Moreover, in [41], researchers claim that they achieved fast protein similarity search tool for short reads. Other researches in [42] employed 53 AD, 53 NC, and also 237 MCI subjects. The authors employed the labeled data for both the AD and NC and also MCI as unlabeled data and projected disease, and they could achieve 53.3%.

In addition, other researchers [1] could achieve 79.4%, with goal of extradomain information that learned from AD and NC. Another researcher [43] employed AD and NC subjects as labeled data and also MCI subjects as unlabeled data and predicted disease labels for MCI cases. In all of these researches, development in the predictive performance of the model was great over supervised learning. Furthermore, by using voxel-stand-D GM features and SVM classifier that other researchers used in 2011 [1], we succeeded to achieve 70.40% for pMCI vs. sMCI by having 76 pMCI and 144 sMCI patients. While the GM feature of ROIs and SVM classifier achieved the classification accuracy of 62% for pMCI vs. sMCI on 43 pMCI and 48 sMCI patients in [1]. Actually, Wolz et al. [44] could achieve 68% of pMCI vs. cMCI on 167 pMCI and also 238 sMCI patients by using LDA classification and used them from four MR features. However, other scientists in 2011 [45] succeeded to achieve 72% for the classification of pMCI vs. sMCI and 86% for AD vs. NC classification of subjects by use of 54 pMCI and 115 sMCI of MR images.

In 2013, Gaser et al. [46] established BrainAGE would be constructed on MRI data for approximating ages of subjects; moreover, mentioning the differences among actual and also estimated age, subjects would be classified into the pMCI or sMCI groups. Besides, they also displayed BrainAGE overtook all cognitive measures and also CSF biomarkers in predicting the conversion of MCI to the AD within 3 years of development. Meanwhile, in 2013, Eskildsen et al. [47] likewise studied MRI biomarker predictive performance in the MCI patients by separating pMCI subjects to the several individuals, in other words, pMCI24, pMCI12, and also pMCI36, and then, they estimated MRI biomarker enactment in every set disjointedly. As a fact, in 2013, Casanova et al. [48] used 188 of NC subjects with 171 of AD, 153 pMCI, and 182 sMCI patients, and they could achieve 62% for AD vs. NC classification, while Min et al. in [49] by using data-driven ROI could obtain 73% for pMCI vs. sMCI by employing 117 pMCI and 117 sMCI cases. Tong and Gao in [50] succeeded to obtain 76% for AD vs. NC classification. Moreover, Moradi et al. [24] have employed 53 AD, 53 NC, and 237 MCI via applying LDS classifier and have used these combining MRI data with cognitive test results MRI and could achieve 61% to the pMCI vs. AD classification. However, at 2016, Ye et al. [51] also employed 51 AD, 52 NC, and 99 MCI patients and have succeeded to attain ACA of AD vs. NC to 87.26% and also pMCI vs. NC to 68.02% and 53.68% for the pMCI vs. NC. Moreover, the authors in [52, 53] by using 128 NC, 97 AD, 117 pMCI, and also 175 sMCI passes could achieve 79% for the task of classification of AD vs. NC, noting that MRI was used for modality.

Moreover, there is a systematic review of all literature on machine learning of neuroimaging for assisted diagnosis of MCI and dementia from 2006 to the end of 2016 in [54]. Furthermore, in [55], genetic algorithm and feature ranking was used for analyzing structural MR images and prediction of MCI to AD from 1 to three years before clinical diagnoses. Moreover, Haller et al. [56] reviewed the basics of pattern recognition comprising a selection of feature, cross-validation, and classification methods and explained restriction, comprising an individual change in NC cases. Last but not least, based on MRIs, we have proposed a classification technique by combining feature decomposition and KDA for AD diagnosis and predicting MCI to AD patients. The experimental results and also comparison indicate the promising performance of the proposed method.

The proposed method also intends for improving disease clarification in informative identification biomarkers which are associated through AD status. Generally, we examine the features of selected imaging through the proposed algorithm. Note that feature selection would be performed on training data only. In addition, Figures 12–14 show how the proposed algorithm can discriminate AD by applying the *t*-test *before* and *after* feature decomposition on brain MR images, for AD vs. NC, pMCI vs. NC, and pMCI vs. sMCI classification, respectively.

Furthermore, after doing some preprocessing as mentioned before *on MR brain images*, *feature decomposition was applied on* whole MR images of the brain; meanwhile, the *t*-test was applied on those images, and we see that result of those image would signify the features that are more related to AD; so we see that combination of *t*-test and feature decomposition can perform as a good AD biomarker.

## 5. Conclusions

The current paper proposed a technique of classification through combining feature decomposition and KDA for AD diagnosis through the help of MR images. At first, MR images are processed to extract the GM densities as the imaging features. Then, to reduce intraclass feature variations, dictionary learning is applied to decompose the features into the class-specific and non-class-specific components, and only the class-specific component of the features is used for classification. Finally, KDA is applied for mapping high-dimensional space of feature toward a linearly separable one for classification. Additionally, our proposed technique could make available an influential and also effective way for identification of more significant biomarkers for classification of brain disease. The proposed technique would be evaluated with MR images of T1-weighted for 830 subjects including 198 AD, 167 pMCI, 236 sMCI, and 229 NC from the ADNI database. The experimental and comparison results indicate the promising performance of the proposed method. Our result in the biomarker also indicates that the proposed scheme could not only be used as a good biomarker but also could enhance the classification of brain disease.

## Figures and Tables

**Table 1:** Demographic characteristics of the standard subjects from ADNI database.

Diagnosis	Number	Age	Gender (M/F)	MMSE (mini-mental state examination)
AD	198	57.5 ± 7.7	103/95	23.3 ± 2.0
NC	229	76.0 ± 5.0	119/110	29.1 ± 1.0
pMCI	167	74.9 ± 6.8	102/65	26.6 ± 1.7
sMCI	236	74.9 ± 7.7	158/78	27.3 ± 1.8

**Table 2:** Results comparison of the feature decomposition, KDA, and also the proposed technique for classification of pMCI vs. NC.

Methods	ACC (%)	SEN (%)	SPE (%)	AUC (%)
Feature decomposition	82.68	76.9	86.88	83.59
KDA	72.59	55.88	84.78	77.91
Proposed method	84.29	90.4	79.85	83.54

**Table 3:** Results comparison of the feature decomposition, KDA, and the proposed method for classification of AD vs. NC.

Methods	ACC (%)	SEN (%)	SPE (%)	AUC (%)
Feature decomposition	88.08	80.87	94.31	92.73
KDA	88.07	80.84	94.31	93.42
Proposed method	90.41	84.37	95.63	93.89

**Table 4:** Results comparison of the feature decomposition, KDA, and the proposed method for classification of pMCI vs. sMCI.

Methods	ACC (%)	SEN (%)	SPE (%)	AUC (%)
Feature decomposition	63.46	45.45	76.66	61.32
KDA	63.45	49.29	73.78	68.22
Proposed method	65.94	81.44	54.69	71.02

**Table 5:** Results of the proposed method comparison and other methods for AD vs. NC.

Methods	ACC (%)	SEN (%)	SPE (%)	AUC (%)
LDA	87.60	82.89	91.64	92.89
SRC [5]	87.83	80.84	93.85	89.77
SVM_1_ [5]	84.57	72.82	94.76	91.40
Proposed method	90.41	84.37	95.63	93.83

**Table 6:** Results of proposed method comparison and other methods for pMCI vs. NC.

Methods	ACC (%)	SEN (%)	SPE (%)	AUC (%)
LDA	83.64	78.56	87.33	81.54
SRC [5]	81.23	83.15	79.79	83.27
SVM_2_ [5]	83.43	88.76	79.50	83.75
Proposed method	84.29	90.4	79.85	83.54

**Table 7:** Results of the proposed method compared with other techniques for pMCI vs. sMCI.

Methods	ACC (%)	SEN (%)	SPE (%)	AUC (%)
LDA	63.31	63.04	70.84	65.80
SRC [5]	64.68	63.87	65.21	66.18
SVM_1_ [5]	64.08	74.35	56.59	69.94
Proposed method	65.94	81.44	54.69	71.02^1^

^1^It would be a radial basis (BF) kernel SVM.

**Table 8:** Comparison between the proposed classification and previous results.

Methods	Subjects	Modalities	AD vs. NC (%)	pMCI vs. sMCI (%)	pMC vs. NC (%)	pMCI vs. AD (%)
Baseline and also longitudinal patterns of the brain [40]	27 pMCI, 76 sMCI	MRI	—	81.5	—	—
Pattern classification using baseline measurements [42]	53 AD, 53 NC, 237 MCI	MRI	—	—	—	53.3
Voxel_stand_D GM features and SVM classifier [1]	76 pMCI, 134 sMCI	MRI	—	70	70.40	—
ROI GM feature and via SVM classifier [43]	51 AD, 52 NC, 99 MCI	MRI	62			
ROI GM feature and via SVM [44]	198 AD, 231 NC, 167 pMCI, 238 sMCI	MRI		64.68	82.76	—
Koikkalainen et al. [45]	54 pMCI, 115 sMCI	MRI	86	72	—	—
BrainAGE framework [46]	188 NC, 171 NC, 133pMCI, 62 sMCI	MRI	—	75	—	—
Separating pMCI subjects from different individuals [47]	61 pMCI, 134 sMCI	MRI		66.7		
Casanova et al. [48]	188 NC, 171AD, 153 pMCI, 182 sMCI	MRI	81.4	61.5	63	—
Data-driven ROI [49]	97 AD, 128 NC, 117 pMCI, 117 sMCI	MRI	—	73.69	—	—
Tong and Gao [50]	191 AD, 229 NC, 161 pMCI, 100 sMCI	MRI	76	—	—	—
Combining MRI data with cognitive test results MRI [24]	53 AD, 53 NC 237 MCI	MRI	—	—	—	61
Discriminative multitask feature selection method [51]	51AD, 52 NC, 99 MCI	MRI	87.2	53.68	68.02	—
Inherent structure-based multiview learning method [52]	97AD, 128 NC, 117 pMCI, 175 sMCI	MRI	92.51	78.88	—	—
Explicitly modeling structural information in the multitemplate data [53]	97 AD, 128 NC, 117 pMCI, 175 sMCI	MRI	93.6	79.25	—	—
**Proposed method**	**98 AD, 229 NC, 167 pMCI, 236 sMCI**	**MRI**	**90.40**	**65.04**	**84.33**	**58.93**

## Data Availability

The MR brain image and clinical data which were used in this study were obtained from Alzheimer's Disease Neuroimaging Initiative (ADNI), publicly available at http://www.adni-info.arg.
